# Supplementary material for: GLP-1R signaling neighborhoods associate with the susceptibility to adverse drug reactions of incretin mimetics
Source: Nat Commun. 2023 Oct 9;14:6243. doi: 10.1038/s41467-023-41893-4 (PMC10562414; doi:10.1038/s41467-023-41893-4)
Supplement: Supplementary file 1 — Supplementary information [file 41467_2023_41893_MOESM1_ESM.pdf]

Supplementary information

GLP-1R signaling neighborhoods associate with the susceptibility to adverse drug reactions of incretin mimetics

Shane C. Wright, Aikaterini Motso, Stefania Koutsilieri, Christian M. Beusch, Pierre Sabatier, Alessandro Berghella, Élodie Blondel-Tepaz, Kimberley Mangenot, Ioannis Pittarokoilis, Despoina-Christina Sismanoglou, Christian Le Gouill, Jesper V. Olsen, Roman A. Zubarev, Nevin A. Lambert, Alexander S. Hauser, Michel Bouvier and Volker M. Lauschke

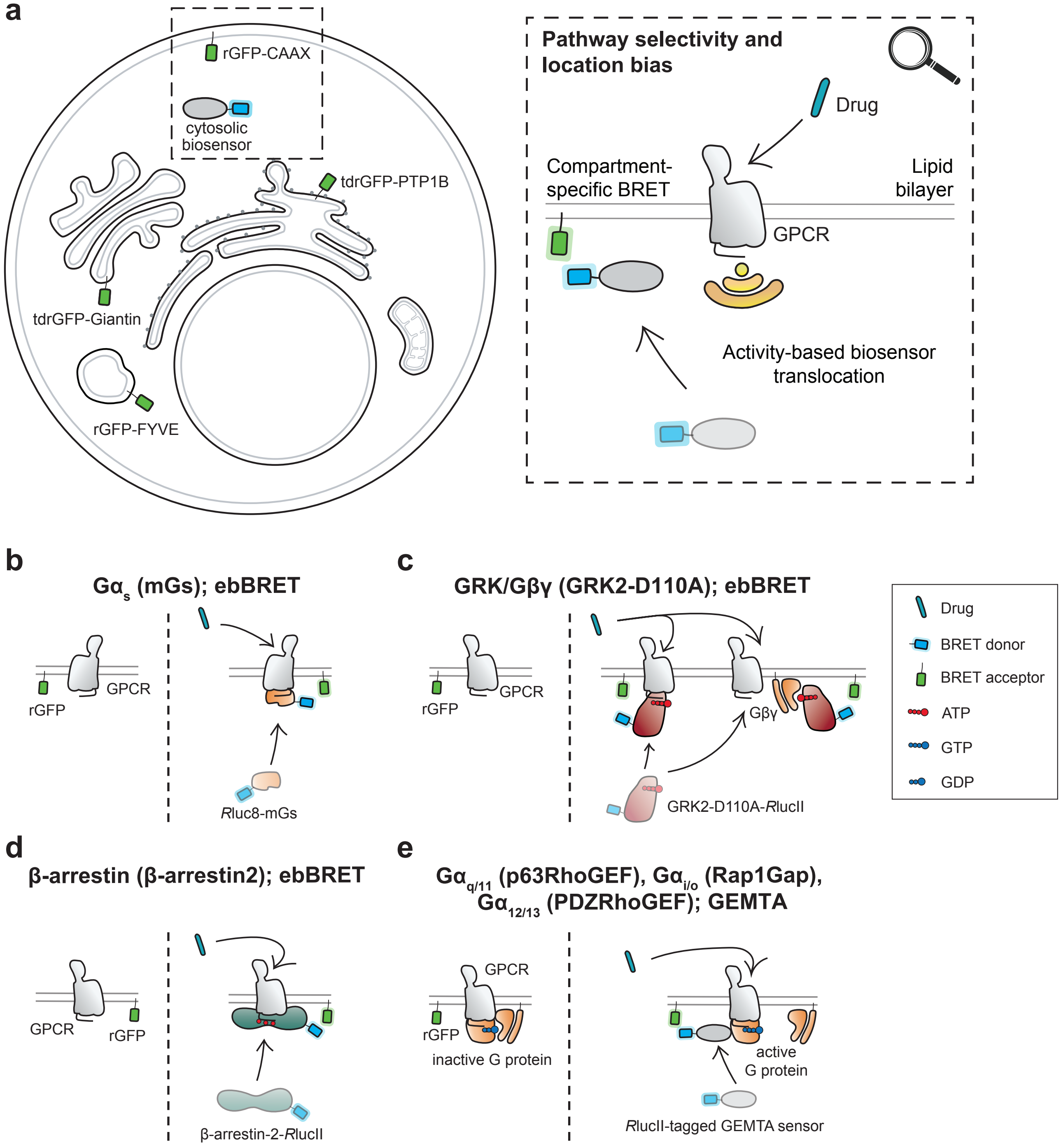
a
rGFP-CAAX
cytosolic
biosensor
tdrGFP-PTP1B
tdrGFP-Giantin
rGFP-FYVE
Pathway selectivity and
location bias
Drug
Compartment-
specific BRET
Lipid
bilayer
GPCR
Activity-based biosensor
translocation
b
Gαs (mGs); ebBRET
GPCR
rGFP
Rluc8-mGs
c
GRK/Gβγ (GRK2-D110A); ebBRET
GPCR
rGFP
Gβγ
GRK2-D110A-RlucII
Drug
BRET donor
BRET acceptor
ATP
GTP
GDP
d
β-arrestin (β-arrestin2); ebBRET
GPCR
rGFP
β-arrestin-2-RlucII
e
Gαq/11 (p63RhoGEF), Gαi/o (Rap1Gap),
Gα12/13 (PDZRhoGEF); GEMTA
GPCR
rGFP
inactive G protein
active
G protein
RlucII-tagged GEMTA sensor

Supplementary Fig. 1. Illustrations depicting the ebBRET-based biosensor platform for investigating GPCR signaling neighborhoods. a, ebBRET monitors *R*luc-tagged biosensor translocation with subcellular resolution through co-expression of organelle-specific markers tagged with rGFP. In this study, signaling neighborhoods were investigated at the (1) plasma membrane – rGFP-CAAX; (2) early endosomes – rGFP-FYVE; (3) Golgi apparatus – tdrGFP-Giantin and (4) endoplasmic reticulum – tdrGFP-PTP1B. **b-e**, Schematics depicting the various ebBRET sensors employed in this study. mGs was used as a proxy for measuring G_s pathway activation by detecting the active receptor conformation that is competent for engaging $G\alpha_s$ (**b**). GRK2-D110A acted as a tool for probing receptor binding or free $G\beta\gamma$ (**c**). β-arrestin2 provided information about either receptor engagement or ligand-directed arrestin trafficking (**d**). GEMTA biosensors measured effector recruitment following G protein activation for the following G protein families: (1) $G_{q/11}$ – p63RhoGEF; (2) $G_{i/o}$ – Rap1Gap or (3) $G_{12/13}$ – PDZRhoGEF (**e**).

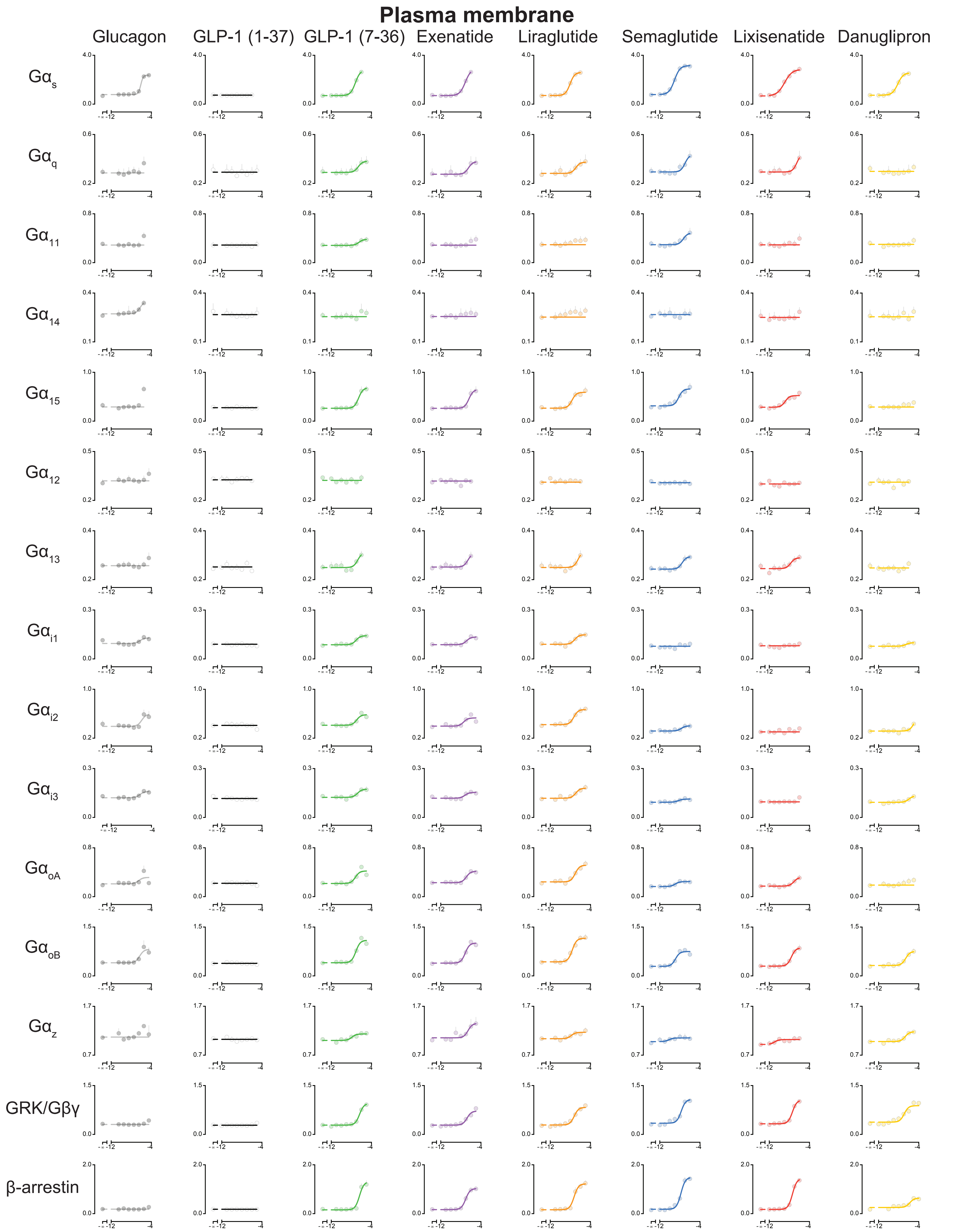

Plasma membrane
Glucagon
GLP-1 (1-37)
GLP-1 (7-36)
Exenatide
Liraglutide
Semaglutide
Lixisenatide
Danuglipron
Gαs
Gαq
Gα11
Gα14
Gα15
Gα12
Gα13
Gαi1
Gαi2
Gαi3
GαoA
GαoB
Gαz
GRK/Gβγ
β-arrestin

Supplementary Fig. 2. Drug-induced transducer engagement by GLP-1R at the plasma membrane. Agonist concentration response curves for 15 transducers at the plasma membrane. Drugs were deemed to activate a given pathway after comparing the top and bottom parameters from non-linear regression by one-sided extra sum-of-squares F-test followed by Bonferroni correction for 8 compounds ($P < 0.00625$). Data are represented as the mean ± SEM (n=3-6 biologically independent experiments). Sigmoidal dose-response curves are present for responsive pathways and flat lines indicate non-responding pathways.

Plasma membrane

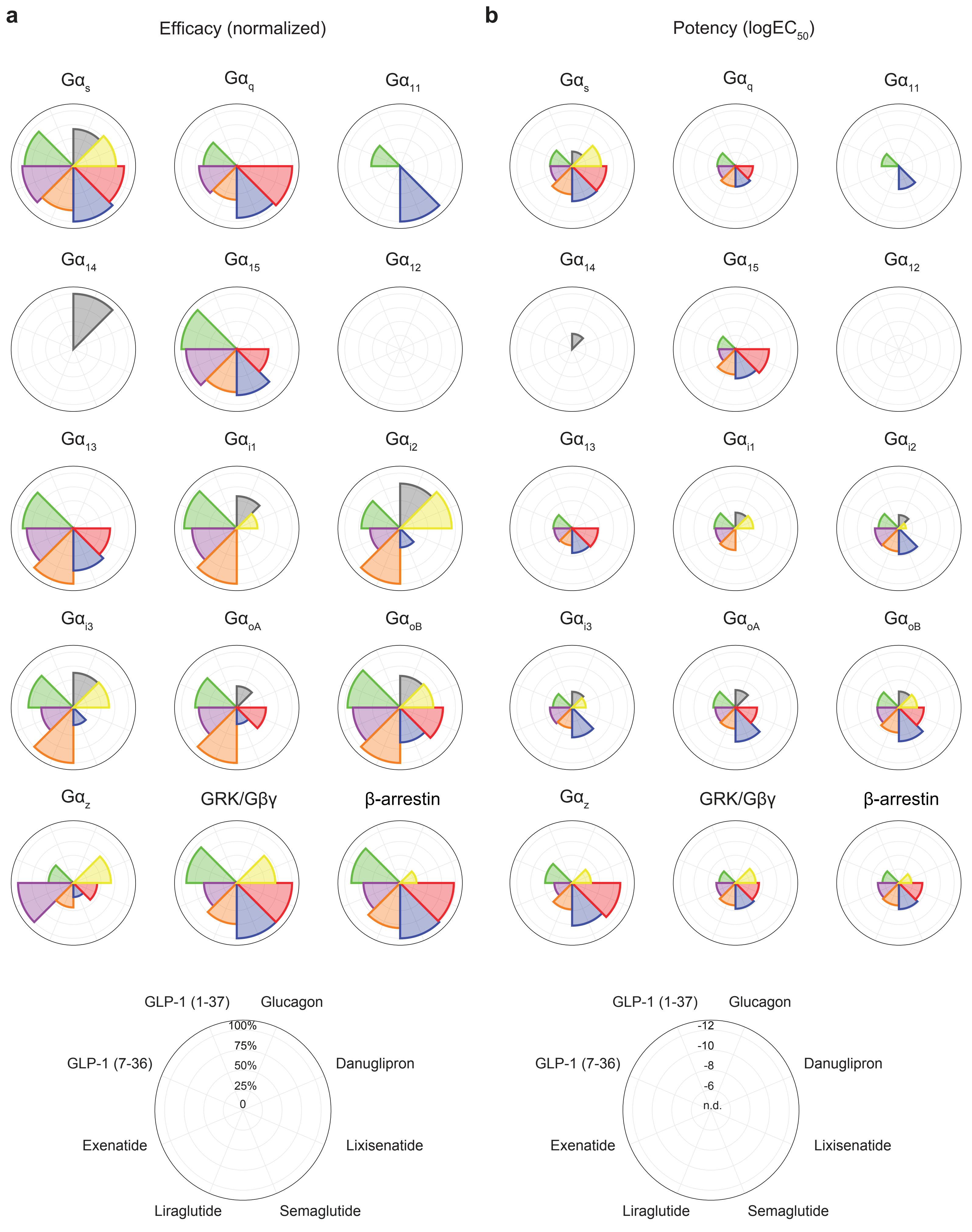

Supplementary Fig. 3. Polar area diagrams depict pathway activation at the plasma membrane. Pathway-specific polar area diagrams for efficacy (normalized to the highest responding drug) (**a**) and potency ($logEC_{50}$) (**b**) of 8 drugs at the plasma membrane. Drugs were deemed to activate a given pathway after comparing the top and bottom parameters from non-linear regression by one-sided extra sum-of-squares F-test followed by Bonferroni correction for 8 compounds ($P < 0.00625$).

mGs translocation to Golgi

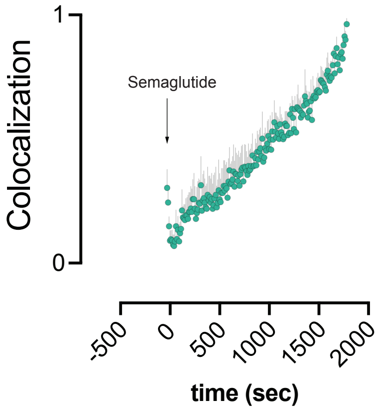

Supplementary Fig. 4. Quantification of colocalization between mGs and Giantin following agonist stimulation. Colocalization between mRuby2-mGs and tdrGFP-Giantin was quantified from timelapse confocal images of HEK293 cells expressing GLP-1R and exposed to semaglutide (1 μM) for 30 min. Data are normalized and represented as the mean ± SEM (*n*=6 cells examined over 3 independent experiments).

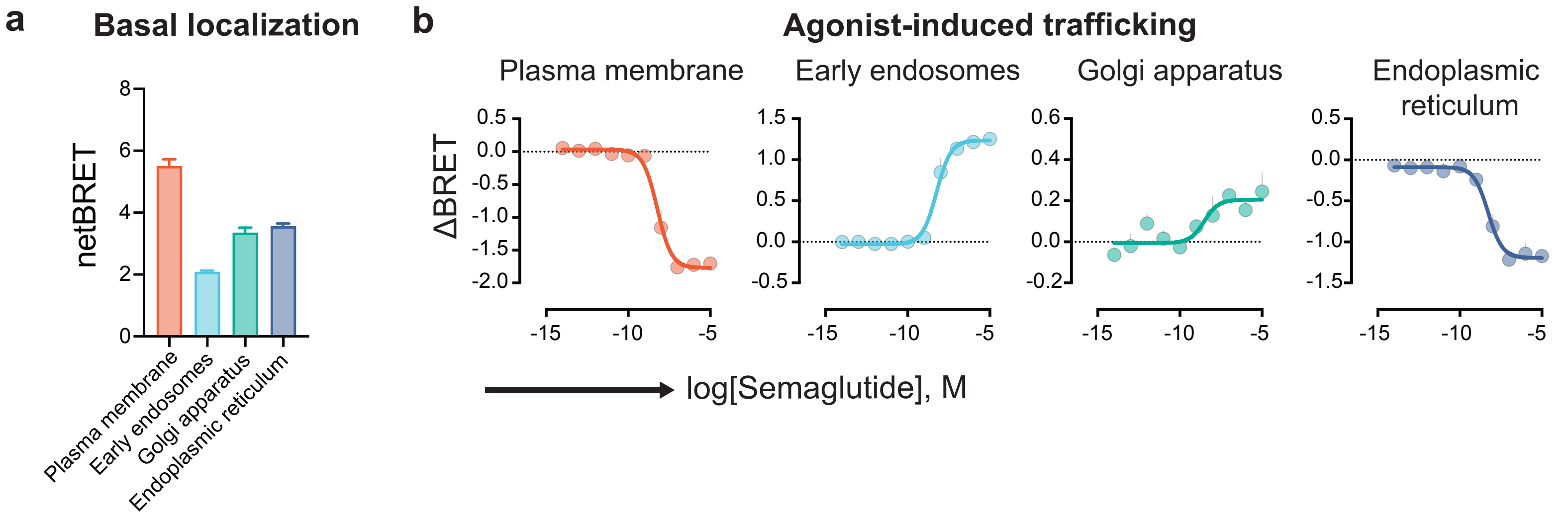
a
Basal localization
netBRET
0
2
4
6
8
Plasma membrane
Early endosomes
Golgi apparatus
Endoplasmic reticulum
b
Agonist-induced trafficking
Plasma membrane
Early endosomes
Golgi apparatus
Endoplasmic reticulum
ΔBRET
0.5
0.0
-0.5
-1.0
-1.5
-2.0
1.5
1.0
0.5
0.0
-0.5
0.6
0.4
0.2
0.0
-0.2
0.5
0.0
-0.5
-1.0
-1.5
-15
-10
-5
log[Semaglutide], M

Supplementary Fig. 5. Basal localization and agonist-directed trafficking of GLP-1R by BRET. **a**, Basal localization of GLP-1R was measured in cells expressing *R*luc8-tagged GLP-1R and rGFP-tagged compartment markers [rGFP-CAAX (PM), rGFP-FYVE (EE), tdrGFP-Giantin (GA) or tdrGFP-PTP1B (ER)]. Data are represented as the mean ± SEM (*n*=3 biologically independent experiments). **b**, Cells expressing *R*lucII-tagged GLP-1R together with rGFP-tagged compartment markers were stimulated with increasing concentrations of semaglutide for the indicated incubation times [PM–15 min; EE–25 min; GA–15 min; ER–25 min]. Data are represented as the mean ± SEM (*n*=3 biologically independent experiments).

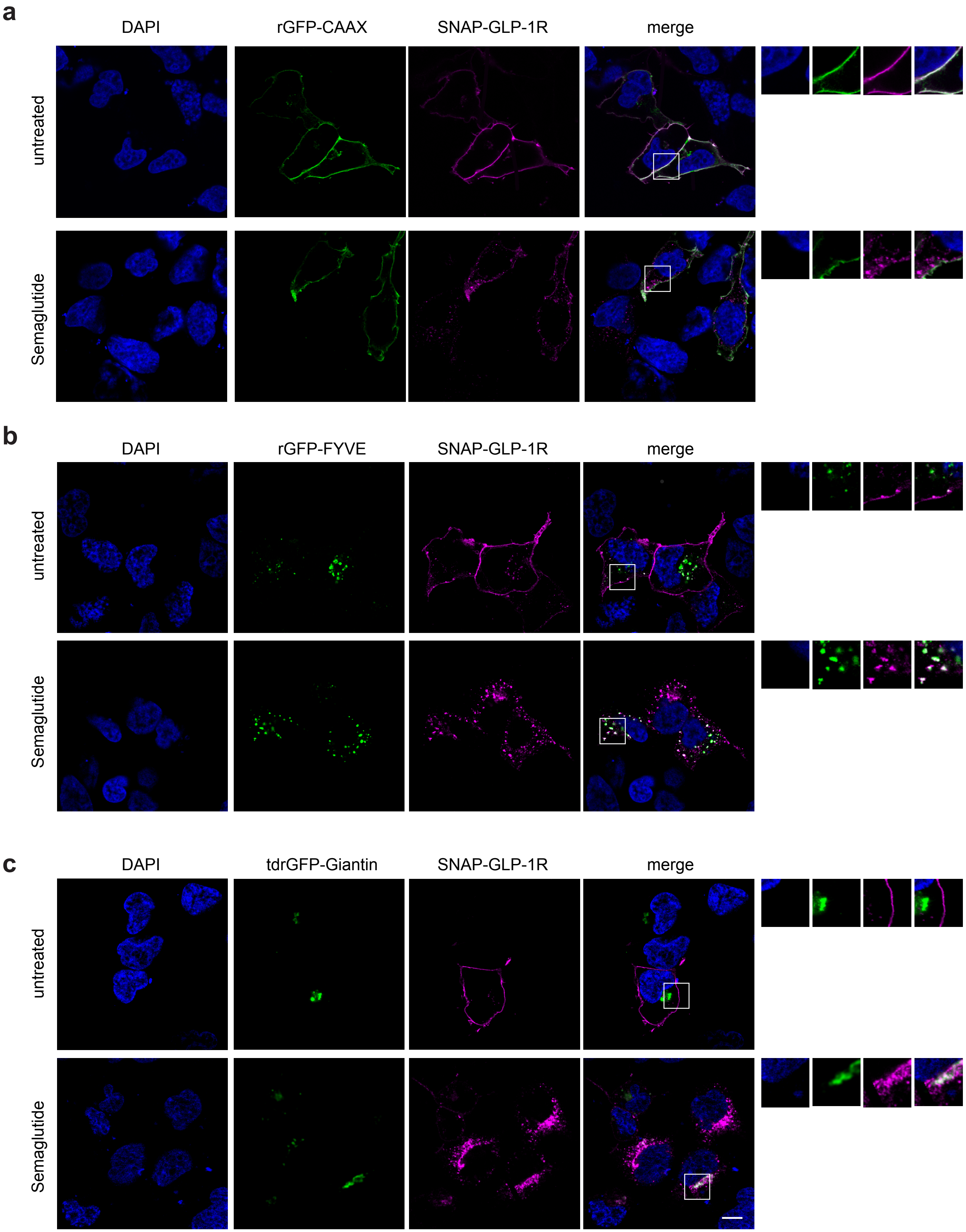
a
DAPI
rGFP-CAAX
SNAP-GLP-1R
merge
untreated
Semaglutide
b
DAPI
rGFP-FYVE
SNAP-GLP-1R
merge
untreated
Semaglutide
c
DAPI
tdrGFP-Giantin
SNAP-GLP-1R
merge
untreated
Semaglutide

Supplementary Fig. 6. Translocation of activated GLP-1R from plasma membrane to early endosomes and Golgi apparatus after semaglutide stimulation. a-c, Confocal images of HEK293 cells expressing SNAP-GLP-1R and rGFP-CAAX (**a**), rGFP-FYVE (**b**) or tdrGFP-Giantin (**c**). Cells were treated with vehicle or semaglutide (1 μM) for 10 min (PM) – 20 min (EE) or 30 min (GA). The presence or absence of colocalization is shown in the inset. Images are representative of 3 independent experiments. Scale bar = 10 μm.

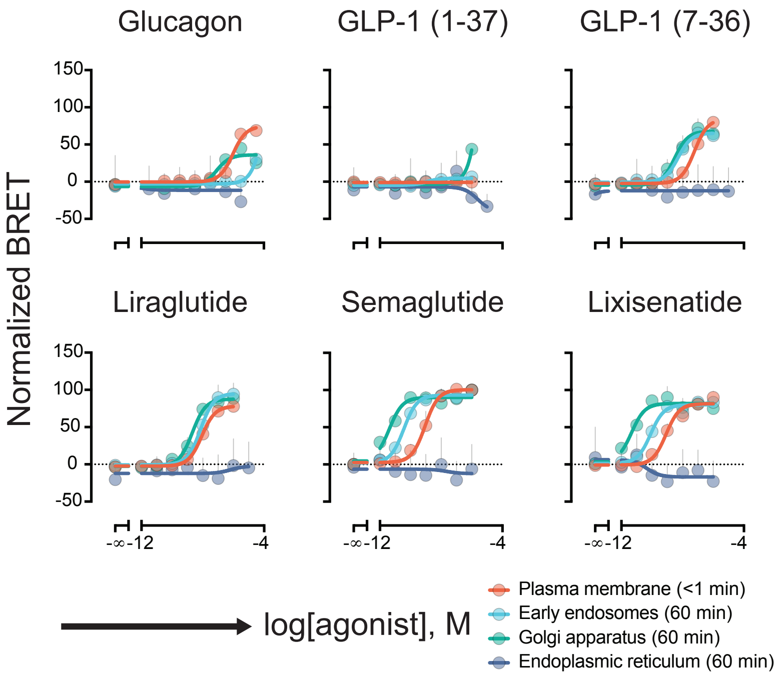
Glucagon
GLP-1 (1-37)
GLP-1 (7-36)
Exenatide
Liraglutide
Semaglutide
Lixisenatide
Danuglipron
Normalized BRET
150
100
50
0
-50
-∞
-12
-4
log[agonist], M
Plasma membrane (<1 min)
Early endosomes (60 min)
Golgi apparatus (60 min)
Endoplasmic reticulum (60 min)

Supplementary Fig. 7. Analysis of G_s pathway engagement by GLP-1R agonists in different compartments. GLP-1R agonist-specific concentration-response curves for G_s pathway activation at the plasma membrane, early endosomes, Golgi apparatus and endoplasmic reticulum. Data are represented as the mean ± SEM (*n*=3 biologically independent experiments).

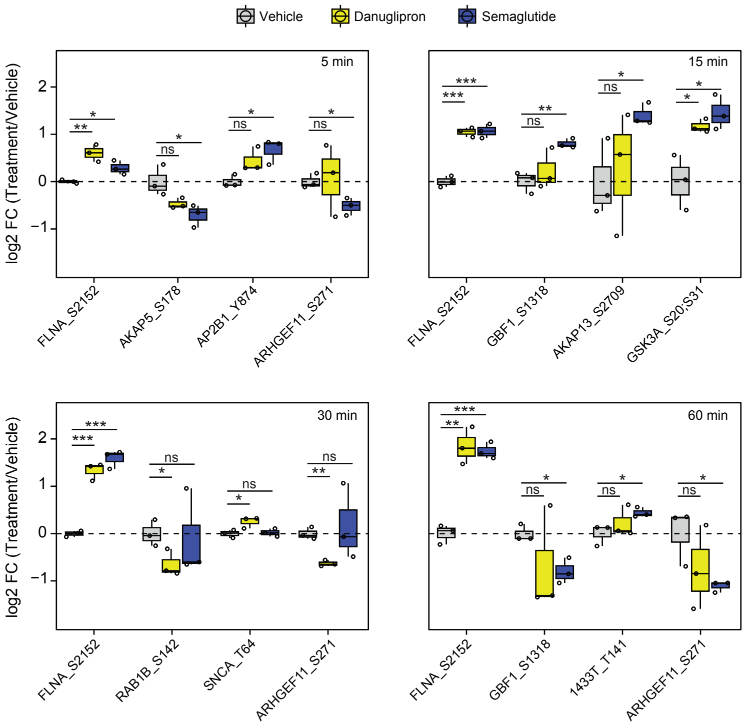
Vehicle
Danuglipron
Semaglutide
log2 FC (Treatment/Vehicle)
5 min
FLNA_S2152
AKAP5_S178
AP2B1_Y874
ARHGEF11_S271
15 min
FLNA_S2152
GBF1_S1318
AKAP13_S2709
GSK3A_S20;S31
30 min
FLNA_S2152
RAB1B_S142
SNCA_T64
ARHGEF11_S271
60 min
FLNA_S2152
GBF1_S1318
1433T_T141
ARHGEF11_S271

Supplementary Fig. 8. Phosphoregulation of proteins downstream of GPCR activation. Box plots of phosphopeptides that are derived from proteins downstream of GPCR activation (UniProt annotation) following exposure to danuglipron or semaglutide at each time point. Phosphorylation sites are annotated beside the protein name (UniProt). Horizontal line in the box plots represents the median, 25th and 75th percentiles and whiskers represent measurements to the 5th and 95th percentiles. $n=3$ biologically independent samples. P-values were calculated using the two-sided Student t-test with equal variance. * $p < 0.05$, ** $p < 0.01$, *** $p < 0.001$.

Early endosomes

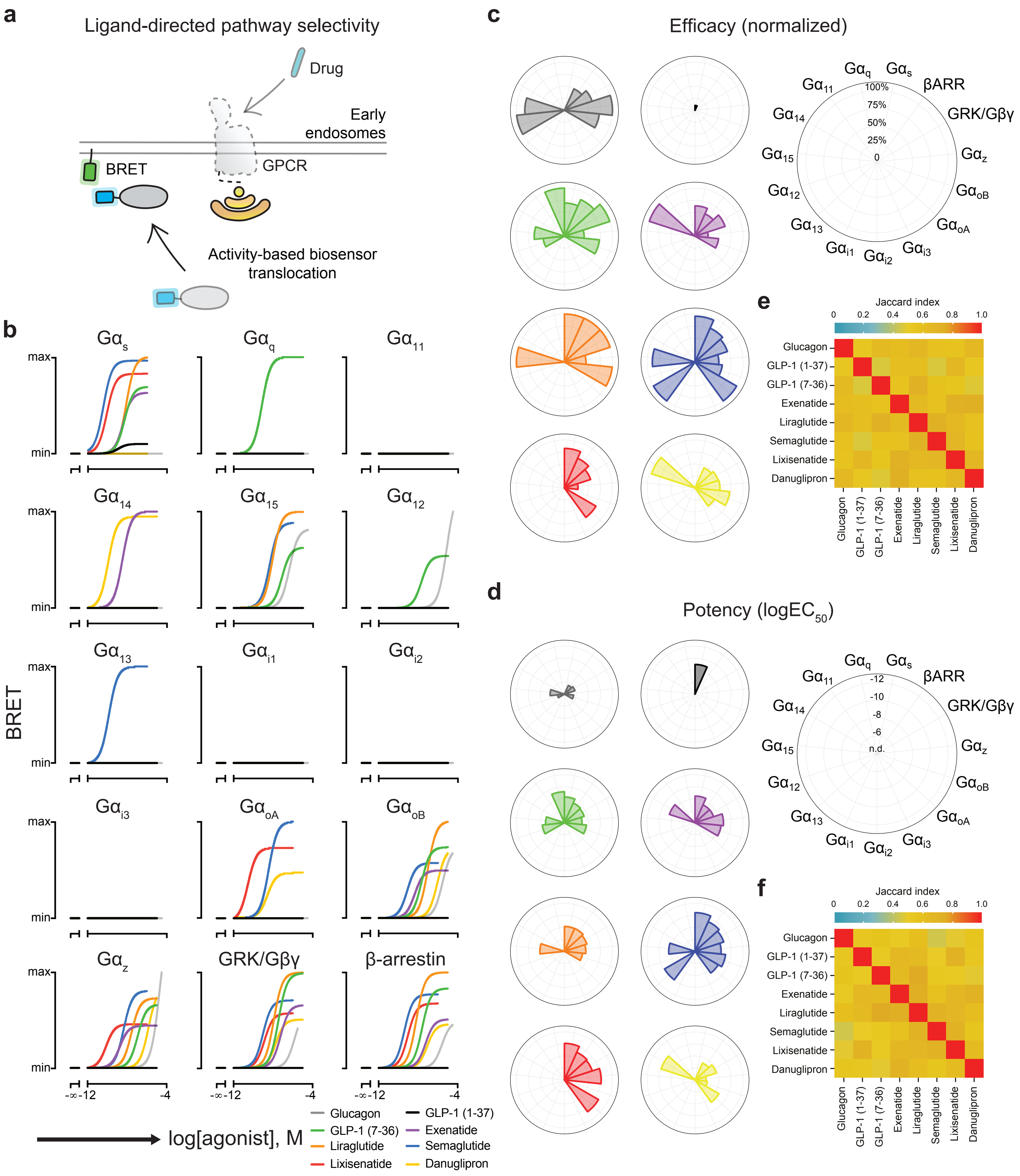

Supplementary Fig. 9. Pharmacological characterization of GLP-1R agonist signaling profiles at early endosomes. **a**, Illustration depicting the ebBRET approach to monitoring signaling at early endosomes. **b**, Concentration-response curves of GLP-1R agonists across 15 pathways at early endosomes using rGFP-FYVE. Data are represented by the non-linear fit and scaled according to the highest responding drug (*n*=3 biologically independent samples). Drug-specific polar area diagrams for efficacy (normalized to the highest responding drug) (**c**) and potency ($logEC_{50}$) (**d**) of 15 pathways at early endosomes. Drugs were deemed to activate a given pathway after comparing the top and bottom parameters from non-linear regression by one-sided extra sum-of-squares F-test followed by Bonferroni correction for 8 compounds ($P < 0.00625$). Jaccard similarity index for efficacy (**e**) and potency (**f**) quantifies similarities and differences across drug responses.

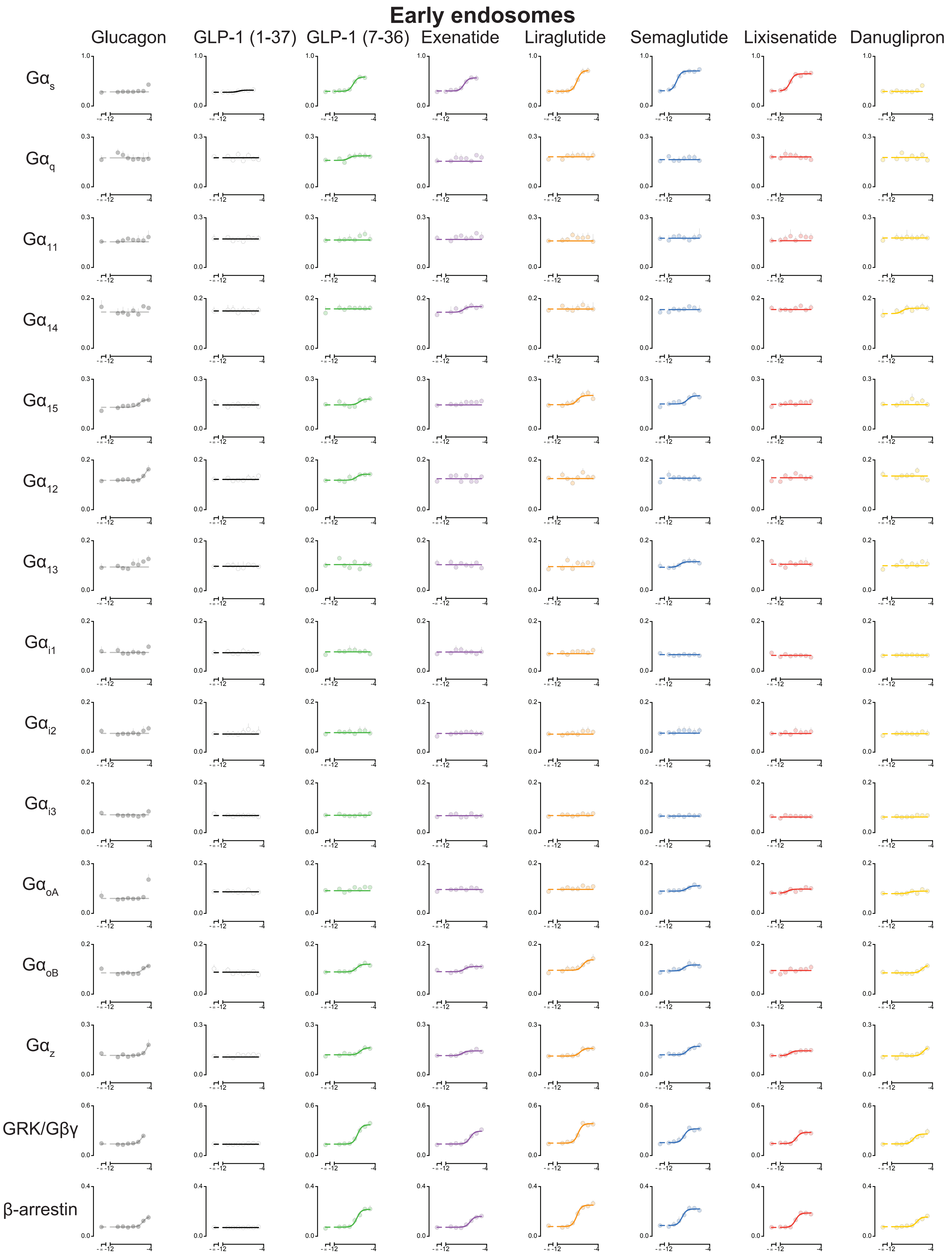

Early endosomes
Glucagon
GLP-1 (1-37)
GLP-1 (7-36)
Exenatide
Liraglutide
Semaglutide
Lixisenatide
Danuglipron
Gαs
Gαq
Gα11
Gα14
Gα15
Gα12
Gα13
Gαi1
Gαi2
Gαi3
GαoA
GαoB
Gαz
GRK/Gβγ
β-arrestin

Supplementary Fig. 10. Drug-induced transducer engagement by GLP-1R at early endosomes. Agonist concentration response curves for 15 transducers at early endosomes. Drugs were deemed to activate a given pathway after comparing the top and bottom parameters from non-linear regression by one-sided extra sum-of-squares F-test followed by Bonferroni correction for 8 compounds ($P < 0.00625$). Data are represented as the mean ± SEM (n=3 biologically independent samples). Sigmoidal dose-response curves are present for responsive pathways and flat lines indicate non-responding pathways.

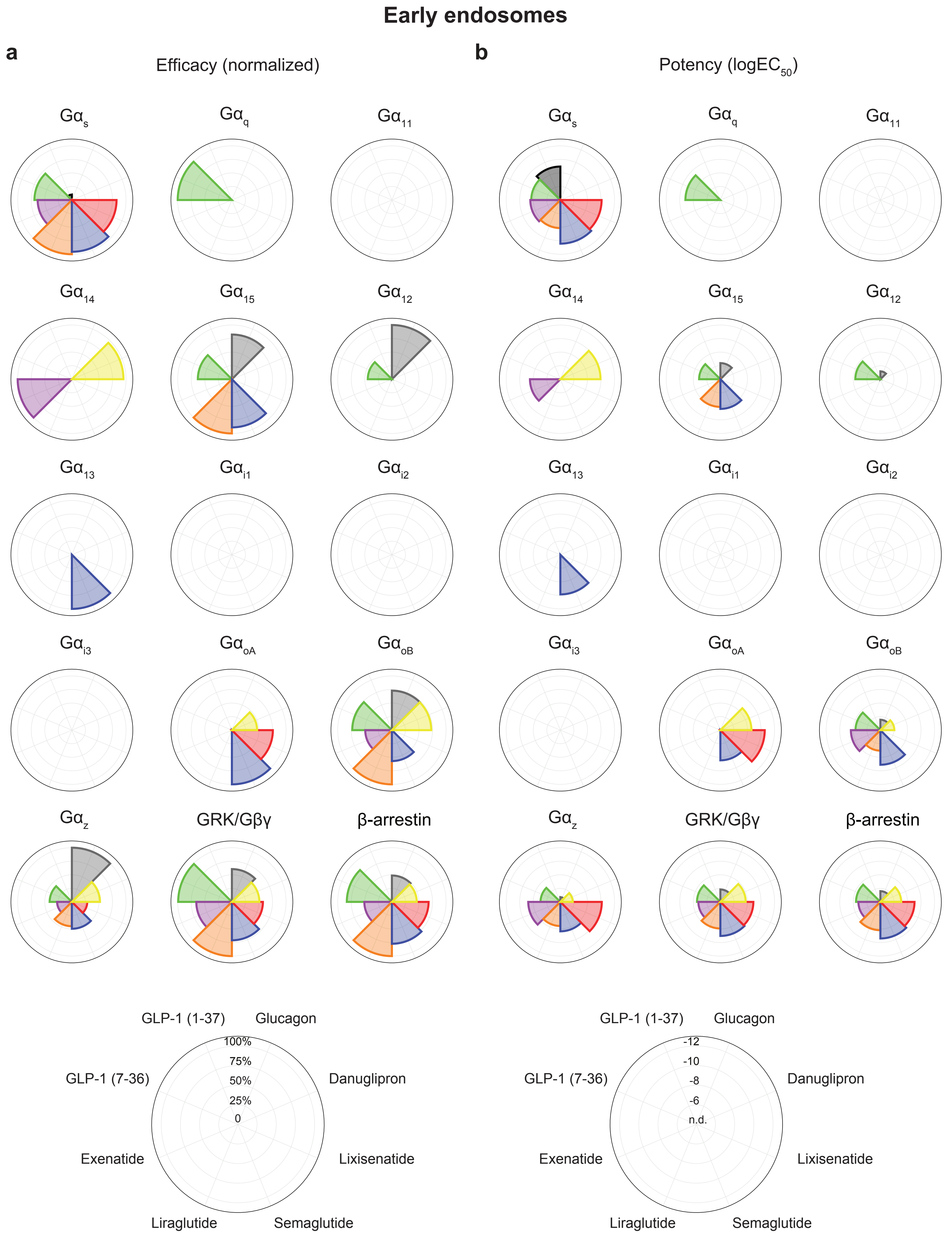
Early endosomes
a
Efficacy (normalized)
b
Potency (logEC50)
Gαs
Gαq
Gα11
Gα14
Gα15
Gα12
Gα13
Gαi1
Gαi2
Gαi3
GαoA
GαoB
Gαz
GRK/Gβγ
β-arrestin
GLP-1 (1-37)
Glucagon
GLP-1 (7-36)
Danuglipron
Exenatide
Lixisenatide
Liraglutide
Semaglutide
100%
75%
50%
25%
0
-12
-10
-8
-6
n.d.

Supplementary Fig. 11. Polar area diagrams depict pathway activation at early endosomes. Pathway-specific polar area diagrams for efficacy (normalized to the highest responding drug) (**a**) and potency ($logEC_{50}$) (**b**) of 8 drugs at early endosomes. Drugs were deemed to activate a given pathway after comparing the top and bottom parameters from non-linear regression by one-sided extra sum-of-squares F-test followed by Bonferroni correction for 8 compounds ($P < 0.00625$).

Golgi apparatus

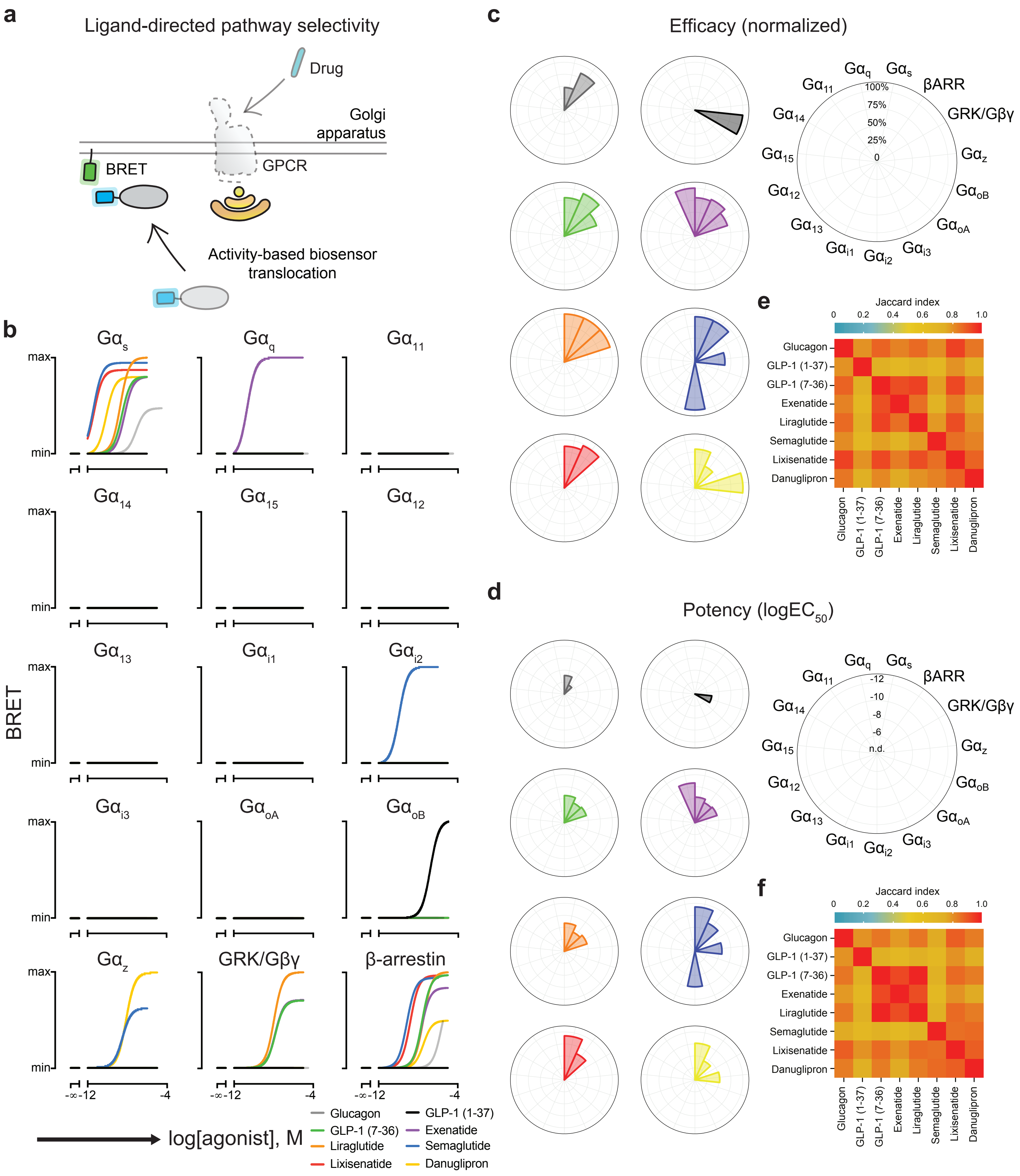

Supplementary Fig. 12. Pharmacological characterization of GLP-1R agonist signaling profiles at the Golgi apparatus. a, Illustration depicting the ebBRET approach to monitoring signaling at the Golgi apparatus. **b**, Concentration-response curves of GLP-1R agonists across 15 pathways at the Golgi apparatus using tdrGFP-Giantin. Data are represented by the non-linear fit and scaled according to the highest responding drug (*n*=3-5 biologically independent samples). Drug-specific polar area diagrams for efficacy (normalized to the highest responding drug) (**c**) and potency ($logEC_{50}$) (**d**) of 15 pathways at the Golgi apparatus. Drugs were deemed to activate a given pathway after comparing the top and bottom parameters from non-linear regression by one-sided extra sum-of-squares F-test followed by Bonferroni correction for 8 compounds ($P < 0.00625$). Jaccard similarity index for efficacy (**e**) and potency (**f**) quantifies similarities and differences across drug responses.

Golgi apparatus

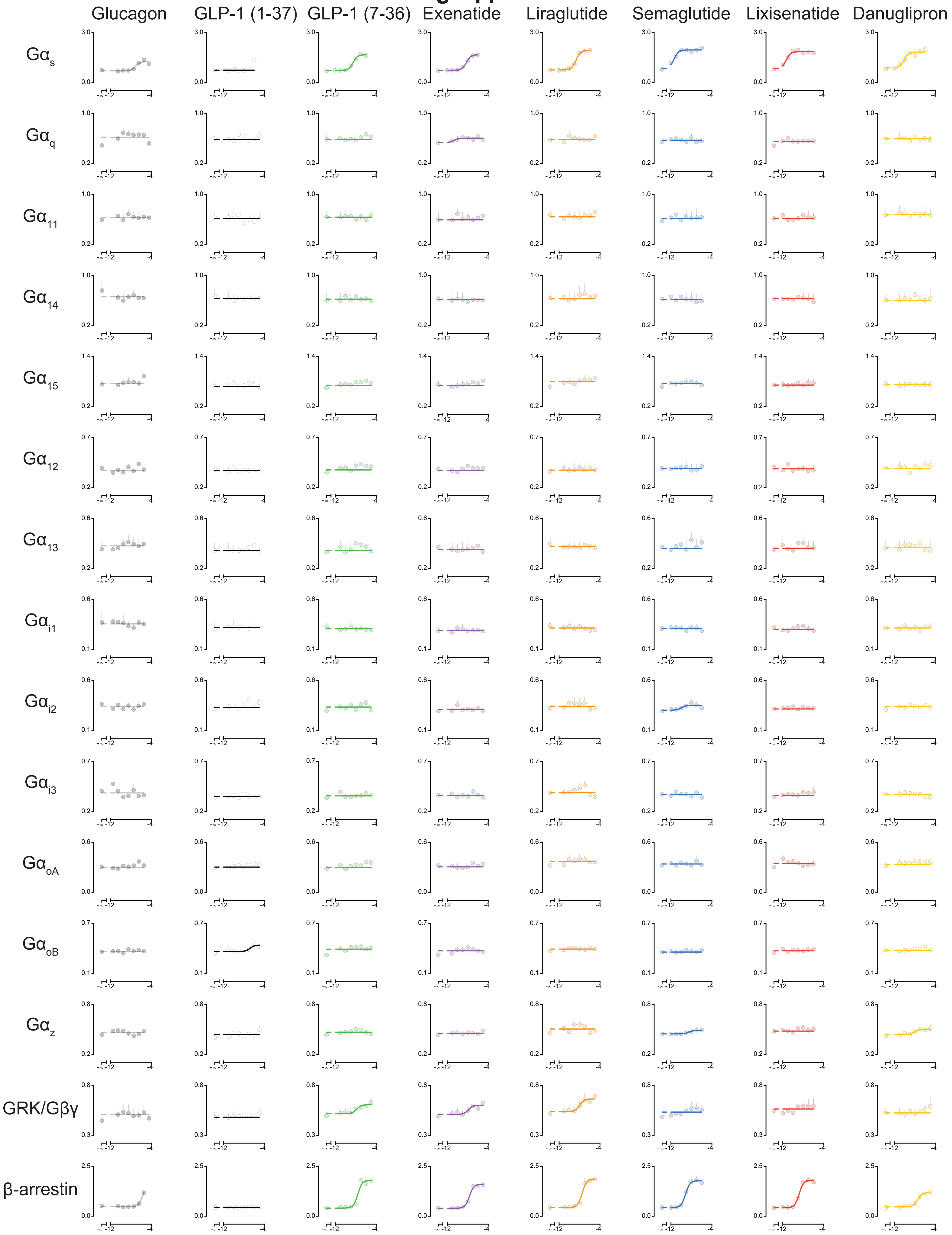

Supplementary Fig. 13. Drug-induced transducer engagement by GLP-1R at the Golgi apparatus. Agonist concentration response curves for 15 transducers at the Golgi apparatus. Drugs were deemed to activate a given pathway after comparing the top and bottom parameters from non-linear regression by one-sided extra sum-of-squares F-test followed by Bonferroni correction for 8 compounds (P < 0.00625). Data are represented as the mean ± SEM (n=3-5 biologically independent samples). Sigmoidal dose-response curves are present for responsive pathways and flat lines indicate non-responding pathways.

Golgi apparatus

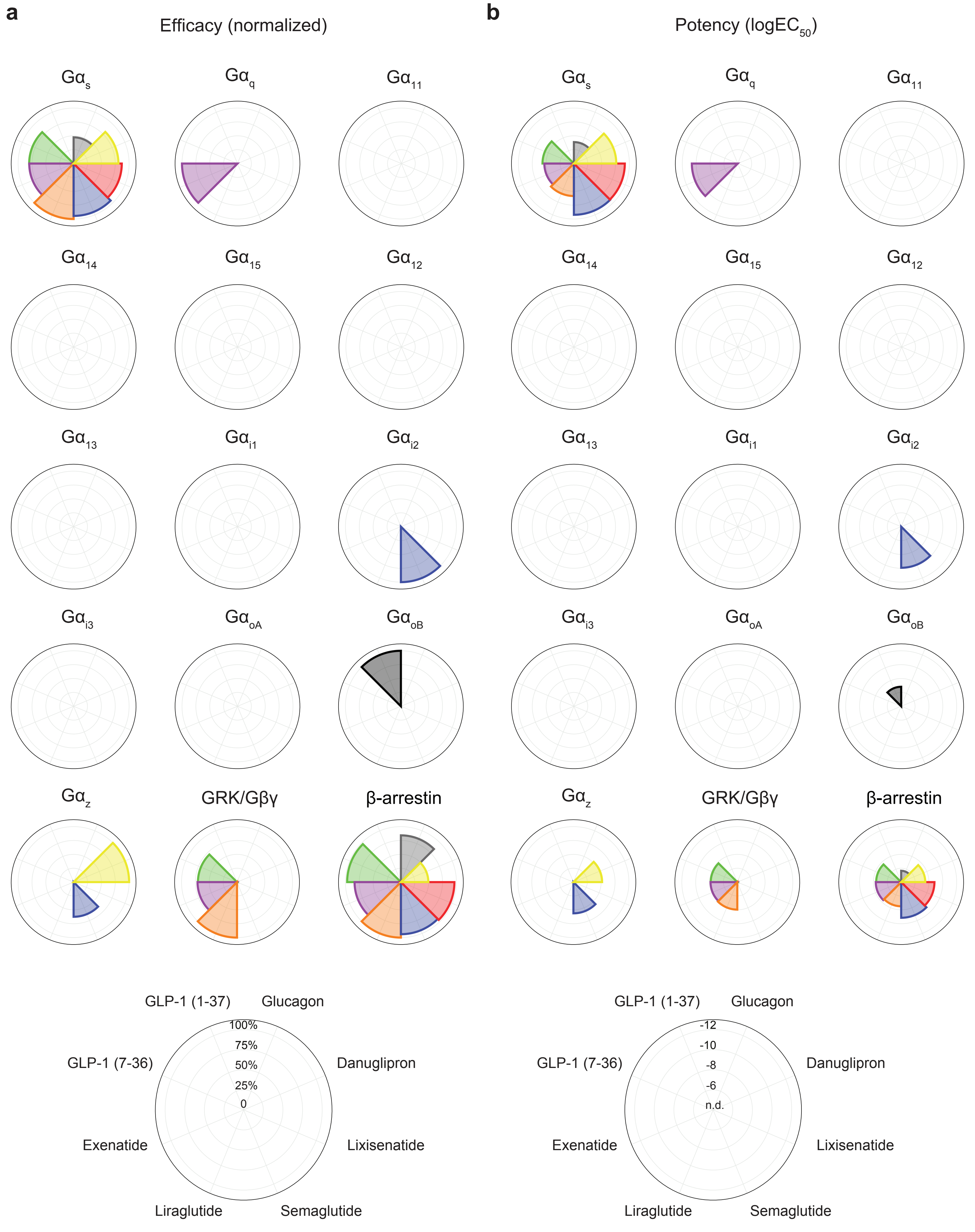

Supplementary Fig. 14. Polar area diagrams depict pathway activation at the Golgi apparatus. Pathway-specific polar area diagrams for efficacy (normalized to the highest responding drug) (**a**) and potency ($logEC_{50}$) (**b**) of 8 drugs at the Golgi apparatus. Drugs were deemed to activate a given pathway after comparing the top and bottom parameters from non-linear regression by one-sided extra sum-of-squares F-test followed by Bonferroni correction for 8 compounds ($P < 0.00625$).

Endoplasmic reticulum

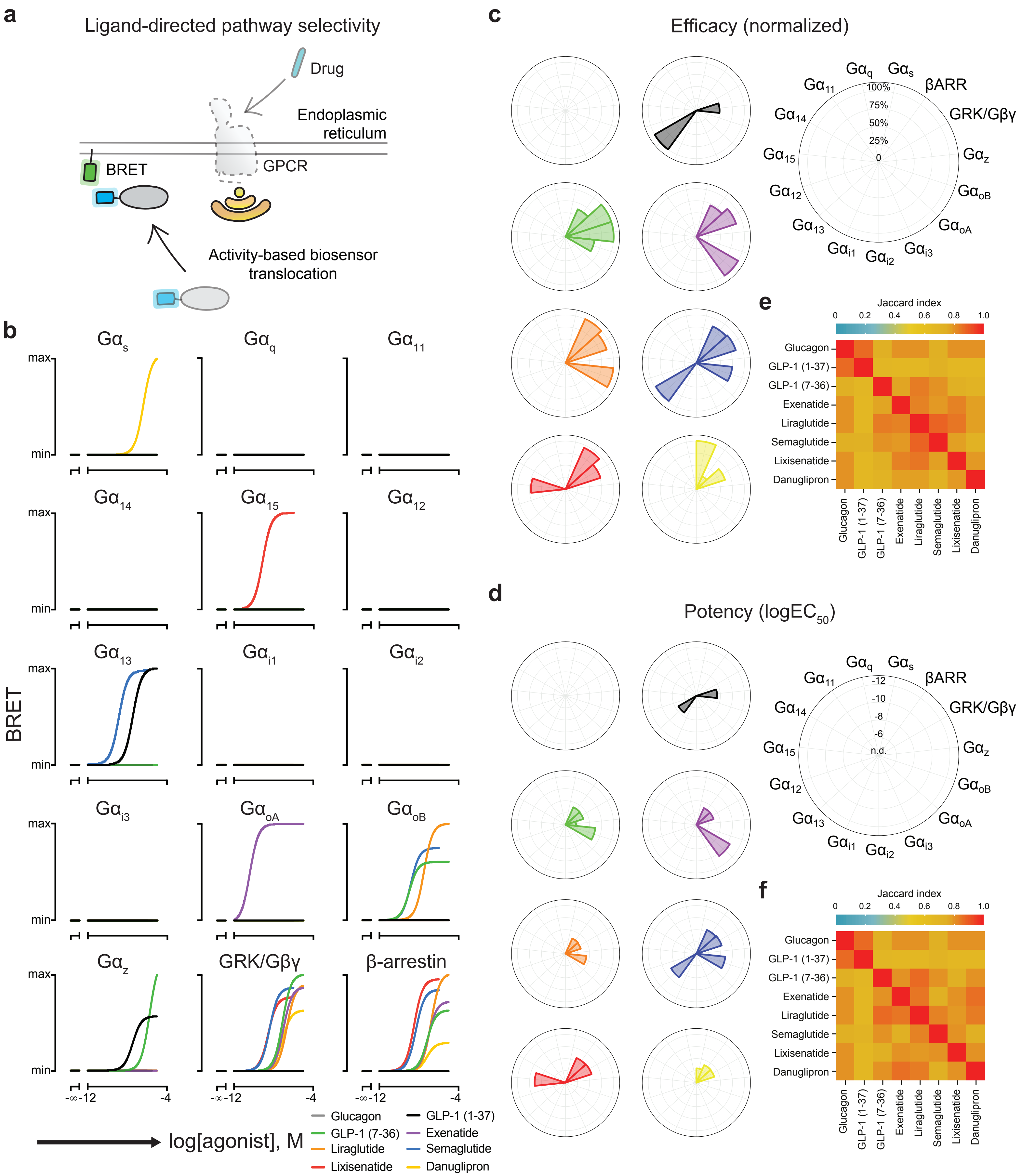

Supplementary Fig. 15. Pharmacological characterization of GLP-1R agonist signaling profiles at the endoplasmic reticulum. a, Illustration depicting the ebBRET approach to monitoring signaling at the endoplasmic reticulum. **b**, Concentration-response curves of GLP-1R agonists across 15 pathways at the endoplasmic reticulum using tdrGFP-PTP1B. Data are represented by the non-linear fit and scaled according to the highest responding drug (n=3 biologically independent samples). Drug-specific polar area diagrams for efficacy (normalized to the highest responding drug) (**c**) and potency ($logEC_{50}$) (**d**) of 15 pathways at the endoplasmic reticulum. Drugs were deemed to activate a given pathway after comparing the top and bottom parameters from non-linear regression by one-sided extra sum-of-squares F-test followed by Bonferroni correction for 8 compounds ($P < 0.00625$). Jaccard similarity index for efficacy (**e**) and potency (**f**) quantifies similarities and differences across drug responses.

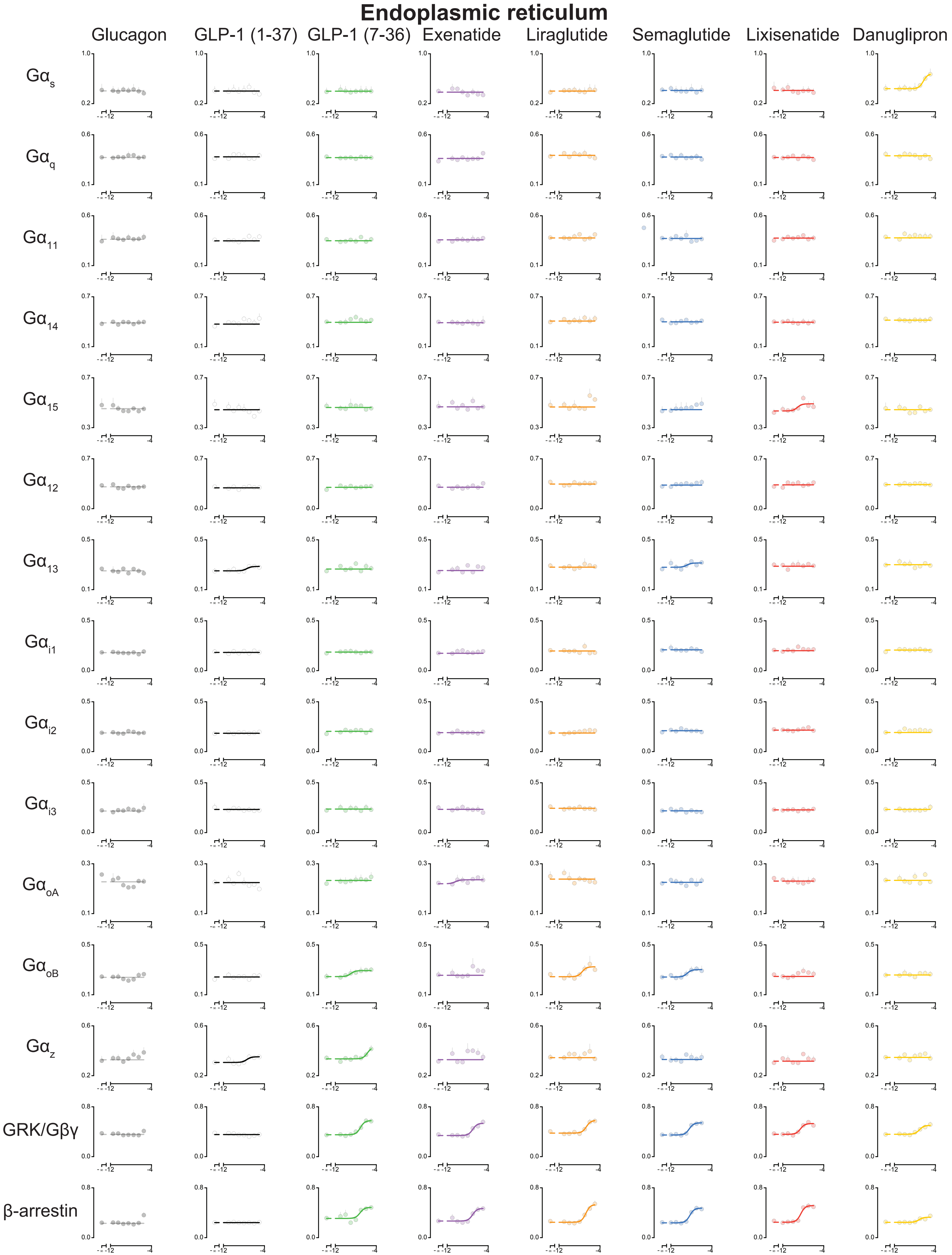
Endoplasmic reticulum
Glucagon
GLP-1 (1-37)
GLP-1 (7-36)
Exenatide
Liraglutide
Semaglutide
Lixisenatide
Danuglipron
Gαs
Gαq
Gα11
Gα14
Gα15
Gα12
Gα13
Gαi1
Gαi2
Gαi3
GαoA
GαoB
Gαz
GRK/Gβγ
β-arrestin

Supplementary Fig. 16. Drug-induced transducer engagement by GLP-1R at the endoplasmic reticulum. Agonist concentration response curves for 15 transducers at the endoplasmic reticulum. Drugs were deemed to activate a given pathway after comparing the top and bottom parameters from non-linear regression by one-sided extra sum-of-squares F-test followed by Bonferroni correction for 8 compounds ($P < 0.00625$). Data are represented as the mean ± SEM (n=3 biologically independent samples). Sigmoidal dose-response curves are present for responsive pathways and flat lines indicate non-responding pathways.

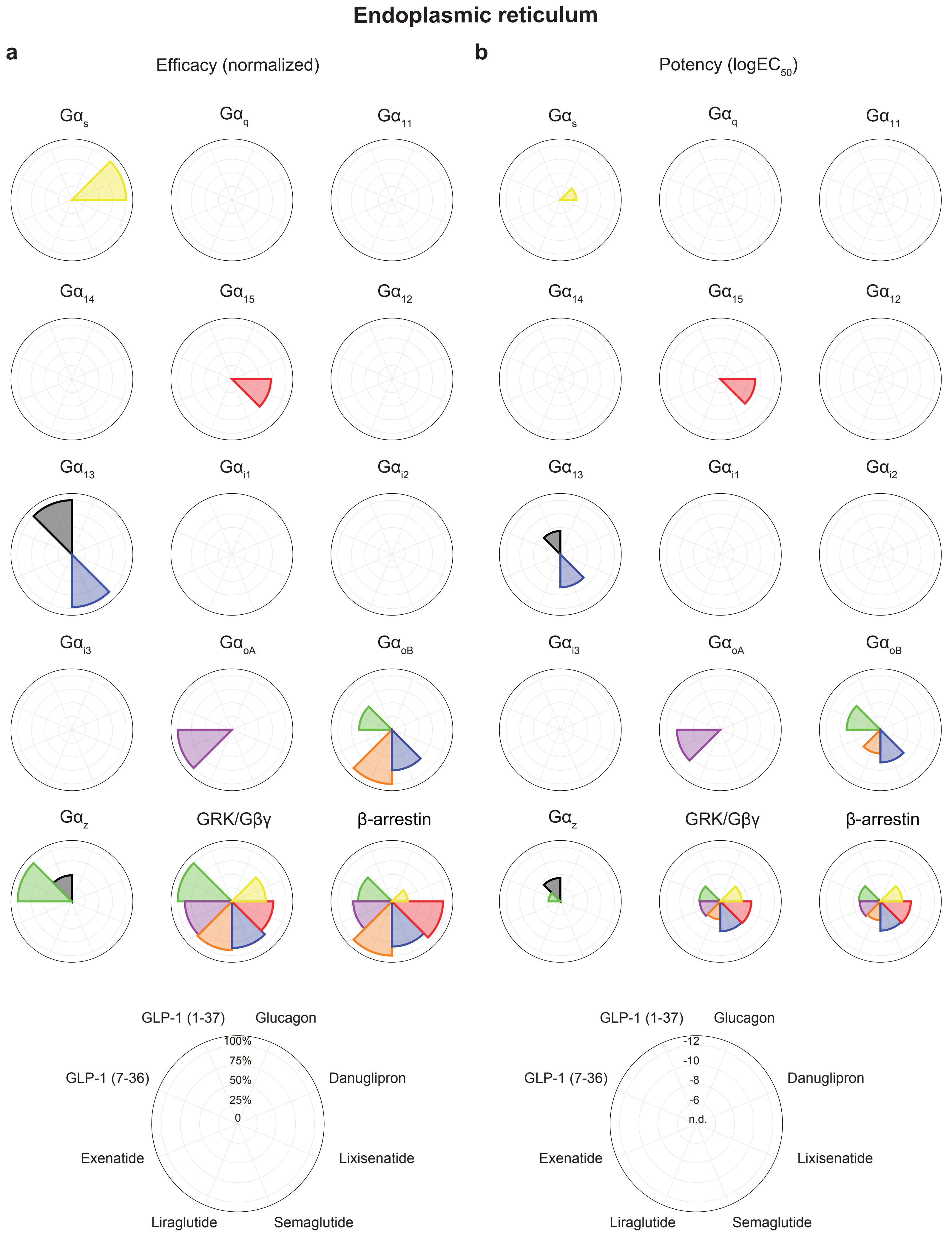

Endoplasmic reticulum
a
Efficacy (normalized)
b
Potency (logEC50)
Gαs
Gαq
Gα11
Gα14
Gα15
Gα12
Gα13
Gαi1
Gαi2
Gαi3
GαoA
GαoB
Gαz
GRK/Gβγ
β-arrestin
GLP-1 (1-37)
Glucagon
GLP-1 (7-36)
Danuglipron
Exenatide
Lixisenatide
Liraglutide
Semaglutide
100%
75%
50%
25%
0
-12
-10
-8
-6
n.d.

Supplementary Fig. 17. Polar area diagrams depict pathway activation at the endoplasmic reticulum. Pathway-specific polar area diagrams for efficacy (normalized to the highest responding drug) (**a**) and potency ($logEC_{50}$) (**b**) of 8 drugs at the endoplasmic reticulum. Drugs were deemed to activate a given pathway after comparing the top and bottom parameters from non-linear regression by one-sided extra sum-of-squares F-test followed by Bonferroni correction for 8 compounds ($P < 0.00625$).

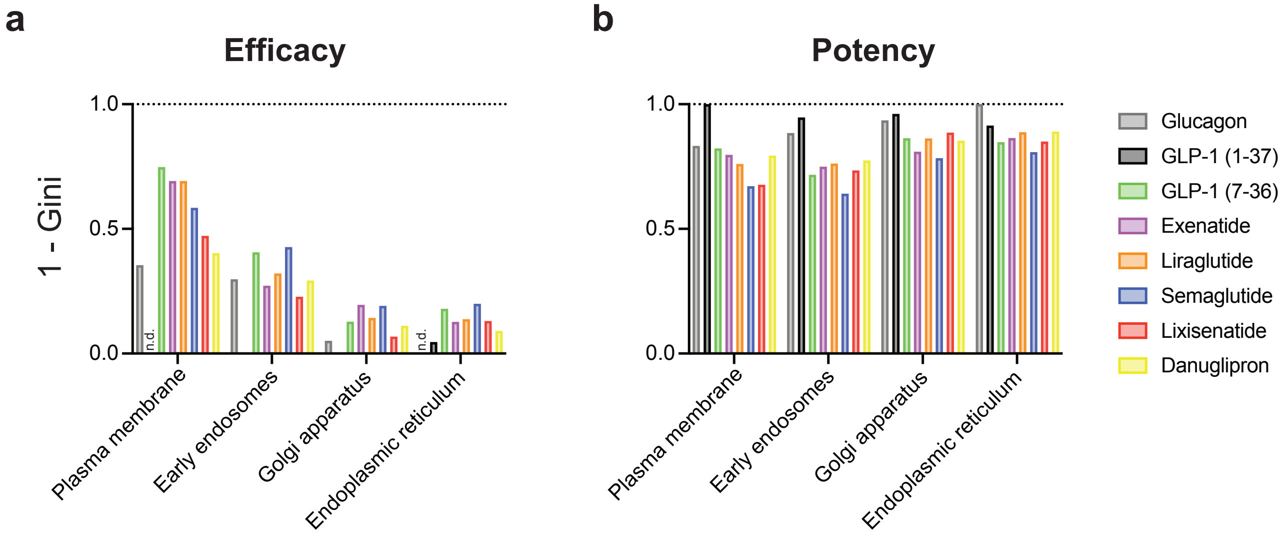
a
Efficacy
1 - Gini
1.0
0.5
0.0
n.d.
n.d.
Plasma membrane
Early endosomes
Golgi apparatus
Endoplasmic reticulum
b
Potency
1.0
0.5
0.0
Plasma membrane
Early endosomes
Golgi apparatus
Endoplasmic reticulum
Glucagon
GLP-1 (1-37)
GLP-1 (7-36)
Exenatide
Liraglutide
Semaglutide
Lixisenatide
Danuglipron

Supplementary Fig. 18. Inverted Gini coefficients depict differences in drug-induced pathway selectivity for efficacy and potency. Inverted Gini coefficients were calculated to compare the similarities among the 15 tested pathways (G_s, G_q, G_{11}, G_{14}, G_{15}, G_{12}, G_{13}, G_{i1}, G_{i2}, G_{i3}, G_{oA}, G_{oB}, G_z, GRK/Gβγ and βARR) for efficacy (**a**) and potency (**b**) across the four compartments included in this study (PM, EE, GA, ER) where 1 represents equality and 0 represents dissimilarity.

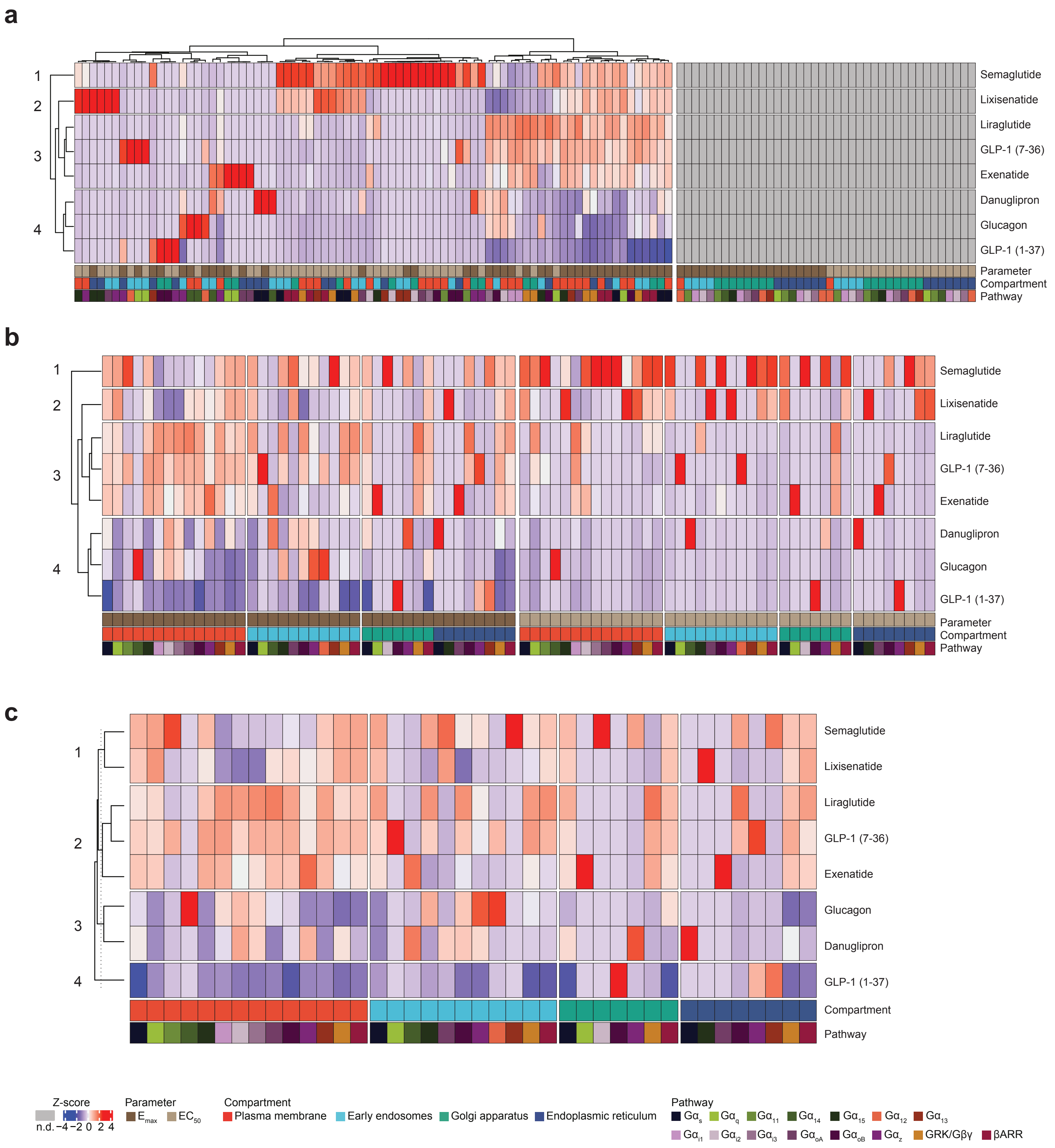
a
1
2
3
4
Semaglutide
Lixisenatide
Liraglutide
GLP-1 (7-36)
Exenatide
Danuglipron
Glucagon
GLP-1 (1-37)
Parameter
Compartment
Pathway
b
1
2
3
4
Semaglutide
Lixisenatide
Liraglutide
GLP-1 (7-36)
Exenatide
Danuglipron
Glucagon
GLP-1 (1-37)
Parameter
Compartment
Pathway
c
1
2
3
4
Semaglutide
Lixisenatide
Liraglutide
GLP-1 (7-36)
Exenatide
Glucagon
Danuglipron
GLP-1 (1-37)
Compartment
Pathway
Z-score
n.d. −4 −2 0 2 4
Parameter
E_{max}
EC_{50}
Compartment
Plasma membrane
Early endosomes
Golgi apparatus
Endoplasmic reticulum
Pathway
$G\alpha_s$
$G\alpha_q$
$G\alpha_{11}$
$G\alpha_{14}$
$G\alpha_{15}$
$G\alpha_{12}$
$G\alpha_{13}$
$G\alpha_{i1}$
$G\alpha_{i2}$
$G\alpha_{i3}$
$G\alpha_{oA}$
$G\alpha_{oB}$
$G\alpha_z$
GRK/Gβγ
βARR

Supplementary Fig. 19. Hierarchical clustering of GLP-1R agonist profiles. a, Heatmap of efficacy and potency for 8 drugs in 15 pathways and 4 cellular compartments. Data are represented as *z*-scores. Pathways that were not activated by GLP-1R upon exposure to ligand are included to the right of the heatmap. Hierarchical clustering was applied to pathways with at least one measured value. **b**, Reductionist representation of heatmap in (a) that is sorted according to efficacy or potency, compartments, and pathways. Pathways that were not activated by any drug were removed for clarity. **c**, Heatmap and hierarchical clustering of efficacy for 8 drugs in 15 pathways and 4 cellular compartments. Data are represented as *z*-scores.

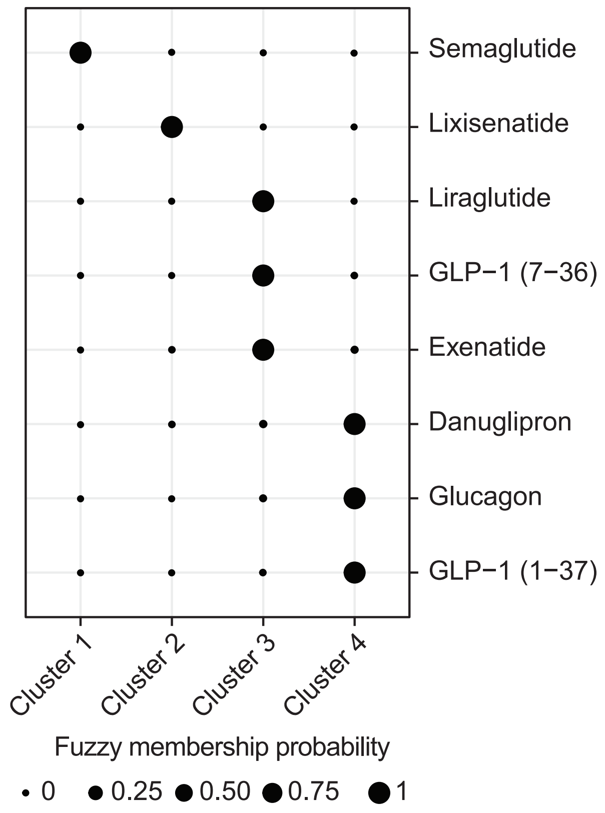
Semaglutide
Lixisenatide
Liraglutide
GLP−1 (7−36)
Exenatide
Danuglipron
Glucagon
GLP−1 (1−37)
Cluster 1
Cluster 2
Cluster 3
Cluster 4
Fuzzy membership probability
0
0.25
0.50
0.75
1

Supplementary Fig. 20. Fuzzy clustering of GLP-1R agonists. Cluster membership probabilities for the GLP-1R agonists were determined based on Fuzzy clustering.

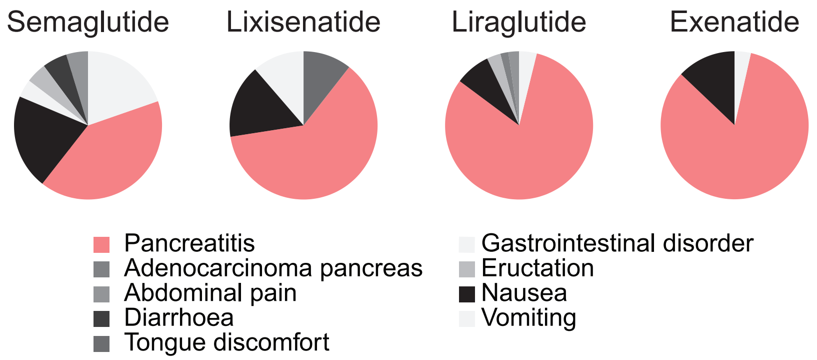
Semaglutide
Lixisenatide
Liraglutide
Exenatide
Pancreatitis
Adenocarcinoma pancreas
Abdominal pain
Diarrhoea
Tongue discomfort
Gastrointestinal disorder
Eructation
Nausea
Vomiting

Supplementary Fig. 21. Pie chart of gastrointestinal disorder likelihood for GLP-1R agonists. The proportion of different ADR gastrointestinal disorder subgroups is shown relative to the overall likelihood. The number underneath each pie chart represents the overall number of events.

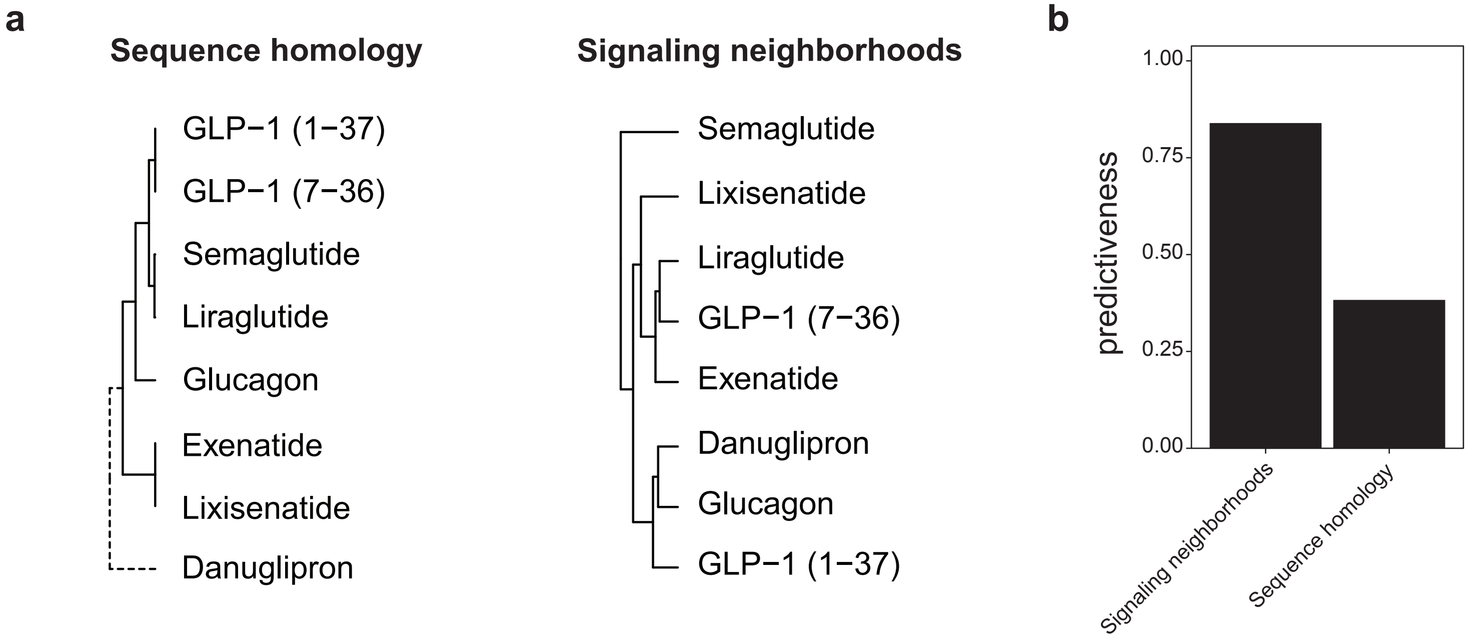

a
Sequence homology
GLP−1 (1−37)
GLP−1 (7−36)
Semaglutide
Liraglutide
Glucagon
Exenatide
Lixisenatide
Danuglipron
Signaling neighborhoods
Semaglutide
Lixisenatide
Liraglutide
GLP−1 (7−36)
Exenatide
Danuglipron
Glucagon
GLP−1 (1−37)
b
predictiveness
1.00
0.75
0.50
0.25
0.00
Signaling neighborhoods
Sequence homology

Supplementary Fig. 22. Dendrograms of GLP-1R agonists based on sequence homology identity and signaling neighborhood profiles. a, GLP-1R agonists were clustered according to their sequence homology or their experimentally determined signaling neighborhoods. Note that danuglipron is a small molecule and is displayed as an outgroup (dotted line) in the sequence homology dendrogram. **b**, Predictiveness of ADRs based on the entanglement score for signaling neighborhoods and sequence homology data.
