# Supplementary material for: GLP-1R signaling neighborhoods associate with the susceptibility to adverse drug reactions of incretin mimetics
Source: Nat Commun. 2023 Oct 9;14:6243. doi: 10.1038/s41467-023-41893-4 (PMC10562414; doi:10.1038/s41467-023-41893-4)
Supplement: Supplementary file 4 — Description of Additional Supplementary files [file 41467_2023_41893_MOESM4_ESM.pdf]

Description of Additional Supplementary files

File name: Supplementary Movie 1.
Description: Semaglutide-induced mGs recruitment to the Golgi apparatus.

File name: Supplementary Data 1.
Description: Structural information for contact pair analysis.

File name: Supplementary Data 2.
Description: Overview of GLP-1R compounds used in the study.

File name: Supplementary Data 3.
Description: List of proteomics samples.

File name: Supplementary Data 4.
Description: Statistical comparisons for Fig. 5b,c,e.

File name: Supplementary Data 5.
Description: Normalized phosphoproteomics data for Fig. 5b,c,e.

File name: Supplementary Data 6.
Description: Statistical comparisons for Fig.5d.

File name: Supplementary Data 7.
Description: Normalized phosphoproteomics data for Fig. 5d.

File name: Supplementary Data 8.
Description: Pharmacological data of GLP-1R agonists across pathways and compartments.

File name: Supplementary Data 9.
Description: Log likelihood ratios of adverse drug reactions for drugs used in the study.

File name: Supplementary Data 10.
Description: Transfection and stimulation conditions for signaling pathway analysis.

File name: Supplementary Data 11.
Description: LC-MS/MS settings.

File name: Supplementary Data 12.
Description: Data-independent acquisition parameters for mass spectrometry.
